# Examining Cardiovascular Disease Risk Profiles Among Older Adults With and Without Fibromyalgia

**DOI:** 10.1093/geroni/igaa057.1388

**Published:** 2020-12-16

**Authors:** Dylan Serpas, Barbara Cherry, Laura Zettel-Watson

**Affiliations:** California State University, Fullerton, Fullerton, California, United States

## Abstract

Introduction: Cardiovascular diseases (CVDs) remain the leading cause of morbidity and mortality in the United States. Preexisting chronic health conditions may be confer increased CVD risk, specifically fibromyalgia (FM), a chronic condition characterized by widespread pain, fatigue, stiffness, and concentration problems. CVD risk increases with normal aging; however, characteristics of FM are suggested to exacerbate health profiles in normal aging processes that may contribute to increased CVD risk. Method: The sample included 221 older adults (M=63.40, SD=8.86; 82% female; 88% White/European American) and 55% reported an FM diagnosis. CVD risk factors were entered separately in a five-block hierarchical binary logistic regression model as predictors and included: cardiorespiratory fitness using the six-minute walk, BMI, standing and lying mean arterial pressure (MAP), and depression using the Beck Depression Inventory. Results: Logistic regression analyses revealed that poorer cardiorespiratory fitness (OR=.99, 95% CI=.99-1.00, p=.001), greater depressive symptoms (OR=1.35, 95% CI=1.19-1.53, p< .001) and lower standing MAP (OR=.98, 95% CI=.96-1.00, p=.036) were associated with higher odds of an FM diagnosis. However, no differences in lying MAP (OR=1.02, 95% CI=1.00-1.04, p=.137) or BMI (OR=1.02, 95% CI=.95-1.10, p=.644) for an FM diagnosis emerged. Discussion: These data support the importance of examining the health profiles of persons with FM in the context of CVD risk. Experiences of FM may produce distinct health profiles with characteristics that serve as both protective and risk factors in the context of CVD.

